# Moving Beyond Somatic Alterations: Uncovering the Germline Basis of Myeloid Malignancies

**DOI:** 10.3390/cancers18020240

**Published:** 2026-01-13

**Authors:** Ismail Elbaz Younes, Lynh Nguyen, Ling Zhang

**Affiliations:** 1 Department of Laboratory Medicine and Pathology, University of Minnesota, Minneapolis, MN 55455, USA; elbaz008@umn.edu; 2 Pathology and Laboratory Medicine, James A. Haley Veterans’ Hospital, Tampa, FL 33612, USA; 3 Department of Pathology, H. Lee Moffitt Cancer Center, Tampa, FL 33612, USA

**Keywords:** germline, predisposition, somatic, mutation, myeloid neoplasm, Down syndrome, RUNX1, CEBPA, DDX41, GATA2

## Abstract

Myeloid neoplasms (MNs) with germline predisposition represent a distinct, increasingly recognized category in the WHO classification, encompassing myelodysplastic syndrome (MDS) and acute myeloid leukemia (AML) arising in the context of an inherited genetic alteration or mutation. While often presenting at a younger age or with characteristic cytopenias with or without organ dysfunction, some can manifest in adulthood, highlighting the need for vigilance regardless of age or family history. Key predisposing genes include transcription factors (e.g., *RUNX1*, *CEBPA*) and genes involved in RNA splicing and telomere biology disorders. Identification of these germline mutations is critical as MNs with germline predisposition dictate specific therapeutic strategies—particularly for hematopoietic stem cell transplantation (HSCT)—and require genetic counseling and surveillance for at-risk relatives. Accurate diagnosis often requires non-hematopoietic germline DNA testing, which provides important biological insights into the development of different myeloid neoplasms and directs personalized patient care.

## Simple Summary

Myeloid neoplasms with germline predisposition develop blood cancers, such as AML and MDS, and arise from inherited genetic mutations rather than acquired ones. Individuals born with errors in genes like *RUNX1*, *GATA2*, or *DDX41* have a significantly increased lifetime risk of developing hematologic malignancies. Identifying hereditary etiology is vital as it influences treatment strategies, including donor selection for stem cell transplant, and necessitates genetic counseling and surveillance for at-risk family members.

## 1. Introduction

Hematologic neoplasms are divided into myeloid and lymphoid neoplasms. Myeloid neoplasms (MNs) include myelodysplastic syndromes (MDSs), myeloproliferative neoplasms (MPNs), MDS/MPNs, and acute myeloid leukemias (AMLs), while lymphoid neoplasms encompass acute lymphoblastic leukemia (ALL) and indolent or aggressive B- or T/NK cell lymphomas. They are derived mostly from somatic mutations or cytogenetic abnormalities. A subset of neoplasms, however, result from germline mutations rather than somatic mutations. Patients carrying these mutations are more prone to develop a myeloid or lymphoid neoplasm. Importantly, germline mutations are constitutional and found in all body cells, including germ cells that can be passed on to offspring, while somatic mutations are acquired after birth and are present in cells excluding germ cells. When determining whether a mutation is germline or somatic, genetic counseling and parallel testing of patients’ familial gene profile are necessary. The development of next-generation sequencing (NGS) has aided in the discovery of these mutations and differentiation between somatic and germline mutations [1].

Historically, the terminology and nature of disease were poorly categorized until recent molecular insights. The incidence of hematologic neoplasms with germline predisposition accounts for 5–10% of hematologic malignancies. The 5th edition of the World Health Organization (WHO) [2] lists myeloid neoplasms with recurrent acquired somatic alterations associated with germline predisposition under secondary myeloid neoplasms that are further subdivided into:

• MNs with germline predisposition without a pre-existing platelet disorder or organ dysfunction;
• MNs with germline predisposition and pre-existing platelet disorders; and
• MNs with germline predisposition and potential organ dysfunction.

The International Consensus Classification (ICC) [3] has similar subcategories for myeloid neoplasms; however, they are not limited to the myeloid lineage. The ICC has an additional category, which includes germline predisposition for lymphoblastic leukemia/lymphoma (ALL) (Table 1). There are several different MNs with germline predisposition under this umbrella (Table 1) that will be described and updated in this review. Myeloid neoplasms associated with bone marrow failure syndromes, myeloid proliferations associated with Down syndrome, and ICC pediatric disorders and/or germline mutation-associated disorders are additional categories related to inherited myeloid neoplasms that have been included in Table 1**,** but are only briefly mentioned and discussed separately. Fanconi anemia, Bloom syndrome, ataxia–telangiectasia, and RASopathies are listed under “genetic tumor syndromes associated with hematolymphoid tumors” in the 5th edition of the WHO and lymphoid neoplasms with germline mutations per ICC will not be focused on in this review.

Importantly, germline predisposition is not considered a disease on its own unless diagnostic criteria for MNs or ALL are met [4]. MNs with germline predisposition were initially discovered in familial platelet disorder with predisposition for AML caused by pathogenic/likely pathogenic germline mutations (P/LP) in *RUNX1* [5,6]. The most important aspect in diagnosing MNs with germline predisposition is the ability to discern if the mutation found is germline or somatic. Germline testing is necessary and can be done by culturing fibroblasts from the skin, buccal mucosa, hair bulbs, saliva, or mononucleated cells from a blood sample, with skin preferred for hematological neoplasms [7]. A variant allele frequency (VAF) between 40–60% (heterozygous) or around 80% (homozygous) is a good indicator of a germline mutation; however, in order to differentiate between somatic and germline mutations, further testing is needed, such as skin fibroblast culture or buccal swab for germline mutations. Disease presentation varies in these MNs and ALL, as some of these mutations are not fully penetrant and should not be relied upon to diagnose MNs or ALL with germline predisposition. Another important factor to consider is age, as some MNs present not only in childhood, but also in adulthood. Therefore, an underlying germline mutation should not be immediately excluded if an MN presents in an adult. Many cases could be missed if one is unaware of this disease category and performs limited molecular investigation. Using larger NGS panels including genes associated with higher cancer risk and germline alterations would be a sensitive tool for screening and identifing these variants. Testing family members is of utmost importance in detecting germline mutations with predisposition for MNs. Closely screening family members can help detect MNs earlier, affording them better prognosis [8].

This review will focus on myeloid neoplasms with germline predisposition according to the 5th edition of the WHO while also being limited regarding lymphoid neoplasms with germline alterations. Clinical significance and underlying mechanisms regarding the disease categories will be updated based on advanced molecular studies.

## 2. Diagnostic Approaches to MNs with Germline Predisposition

It is crucial to establish a diagnostic algorithm for assessment of MNs with germline predisposition and separate them from carriers. Accurate diagnosis of MNs with germline predisposition requires integration of familial history, genetic consultation, and laboratory tests including examination of peripheral blood and bone marrow, NGS, and RNA sequencing. There are some clues when suspecting an underlying germline-mutated MN, including:

• Careful family history, which would show multiple myeloid malignancies at a young age, e.g., MDS in the early 40s, hypoplastic pediatric MDS, or family members carrying clonal molecular and/or cytogenetic abnormalities.
• Congenital physical anomalies or syndromes should also raise suspicion; e.g., Down syndrome and dyskeratosis congenita.
• Congenital immunodeficiency or specific immune cell loss linked to germline gene mutation leading to Bloom or MonoMAC syndrome.
• Related donor-derived MNs postallo-HSCT.
• VAF close to 45–50% or 80–100%; persistently high VAF and/or biallelic mutations warrant careful analysis of a germline process.

Differentiating between somatic and germline mutations is imperative. Bear in mind that detection of a germline mutation is associated with increased risk of myeloid malignancies. WHO or ICC criteria should be followed to diagnose a myeloid neoplasm in a setting of a germline mutation. Additionally, age of onset of a myeloid malignancy is variable and does not exclusively present in childhood. For example, AML with germline *DDX41* mutation often occurs after 70 years of age. Presence of increased blasts or complex karyotype in a peripheral blood and/or bone marrow sample can aid in the diagnosis of MNs. Of note, clonal hematopoiesis of indeterminated potential (CHIP) is not uncommon in patients with germline mutations. Careful differentiation between germline and acquired somatic mutations is necessary before rendering a diagnosis of MDS/AML [9] with specific germline predisposition. It is also critical to look for gene mutations specific to MDS, e.g., *BCOR*, *SF3B1*, *U2AF1*, *EZH2*, *SRSF2*, *ASXL1*, and *ZRSF2*, when performing differentiation.

### 2.1. Myeloid Neoplasms with Germline Predisposition Without a Pre-Existing Platelet Disorder or Organ Dysfunction

This category includes three different genes that are implicated: Germline *CEBPA* P/LP variant (*CEBPA*-associated familial AML), Germline *DDX41* P/LP variant, and Germline *TP53* P/LP variant [10,11,12,13].

#### 2.1.1. Germline *CEBPA* P/LP Variant-Associated Familial AML

CCAAT/enhancer binding protein-α (*CEBPA*) is an intronless protein, functioning as a myeloid transcription factor that is present on chromosome 19q13.1. It encodes two proteins (3p and 42 kD protein) depending on the start site. It is expressed in hepatocytes, adipocytes, type II pneumocytes, follicles, and granulocytic precursors [14,15,16,17]. It plays an especially important role in enhancing granulocytic differentiation toward end-stage neutrophils and monocytes [18,19]. Mutations in *CEBPA* can occur at the C-terminus (frame-shift mutations) or N-terminus (in-frame insertions/deletions) [20]. Mutations that occur at the N-terminus are commonly frameshift or nonsense mutations, are usually germline in origin, and do not meet criteria for AML with in-frame basic region leucine zipper (bZIP) *CEBPA* mutations [3]. Mutations in the C-terminus are usually secondary to somatic mutations in patients with pre-existing germline N-terminus mutations. They also occur in the bZIP domain, which is diagnostic of AML with *CEBPA* mutation. The disease is inherited in an autosomal dominant pattern. The germline mutations encompass both pathogenic and likely pathogenic variants [21] and result in transcriptional dysregulation.

Gunz et al. described 13 family members, spanning three generations, who suffered from leukemia [22]. This phenomenon was not explained until 2010 when the pathogenesis of *CEBPA* germline predisposition was discovered [23]. They found that affected family members carried a base pair deletion of the N-terminal (c.68delC) and another acquired a mutation in the C-terminus. In 2004 Smith et al. reported another family that had three members who carried the same *CEBPA* mutation and developed leukemia [24].

The mutation site appears critical for development of familial *CEBPA*-mutated AML versus sporadic AML with *CEBPA* mutation. Familial *CEBPA*-mutated AML is reminiscent of sporadic AML with *CEBPA* mutation in terms of survival and pathologic features [10]. In this entity, germline mutations do not only occur in the N-terminus. Studies showed that the C-terminus can harbor germline mutations leading to the development of this MN [25]. Patients who had germline mutations in the C-terminus eventually developed AML; however, they did not have a family history of AML. This finding supports that germline mutations in the C-terminus have variable penetrance while germline mutations in the N-terminus have almost complete penetrance with a disease onset of 2–50 years of age [10]. It is further supported that in patients carrying a germline mutation in the N-terminus, the acquisition of another somatic *CEBPA* mutation is needed to develop AML [26]. The molecular profile in patients with germline mutations in the N-terminus of *CEBPA* is similar to that of sporadic cases of AML with double mutations of *CEBPA* [26]. Of note, patients with familial *CEBPA*-mutated AML had better overall survival (OS) than those with sporadic double-mutated *CEBPA* AML, 8 years versus 16 months, suggesting better response to therapy [26]. Additionally, the familial type has a different molecular profile at recurrence, whereas the sporadic counterpart tends to have the same molecular profile at initial diagnosis and recurrence.

Currently, there is no consensus on the appropriate time to test germline mutations of *CEBPA*. Suspicion should be high for a germline predisposition, especially in patients developing AML with mutated *CEBPA* under the age of 50. It is imperative to undergo germline testing in this setting as studies have identified that 5–10% of sporadic *CEBPA*-mutated AML carry an underlying germline mutation [25,27]. 

#### 2.1.2. Germline *DDX41 P/LP* Variant

DEAD-box helicase 41 (*DDX41*) gene is located on chromosome 5q35.3. Though not fully understood, it is thought to play a role in pre-mRNA splicing [28]. In 2020, a study by Tsukamoto et al. revealed that the analog to *DDX41* (*SACY-1*) in *C. elegans* plays a role in splicing [29]. Another study showed that an R525H mutation in *DDX41* altered protein–protein interactions, especially for U2 and U5 spliceosome [30]. *DDX41* is also involved in ribosome biosynthesis and innate immunity by sensing invading nucleic acids [28]. *DDX41* mutations play a role in myeloid neoplasms as a study showed cases of AML associated with somatic *DDX41* mutations [31]. It was not until 2015 that germline mutations in *DDX41* were found to play a role in predisposition to myeloid neoplasms [30]. This was later confirmed when two different patients developed donor cell leukemia after stem cell transplant from donors carrying germline *DDX41* mutations [32,33]. Germline mutations in *DDX41* are the most common mutations that predispose to AML and MDS accounting for 2% of cases [34,34]. In MN with germline-mutated *DDX41*, the most common somatic mutation is another *DDX41* (R525H being the most common mutation), followed by *ASXL1* mutations [34]. A recent study conferred that R525H somatic mutation was the most common mutation found in their cohort of patients with *DDX41* germline mutations [35]. Most of the germline mutations in *DDX41* were either frameshift, nonsense, or splicing site mutations [36,37]. A cohort study carried out in 2021 found that p.M1I, p.D140fs, and p.Q41* were the most common germline mutations [37]. Importantly, MDS patients harboring truncating germline mutations in *DDX41* had a more rapid progression to AML compared to those harboring non-truncating variants, though there was no difference in the overall survival [38]. The somatic and germline mutations in *DDX41* are in different locations. Germline mutations occur upstream of the helicase 2 domain and involve loss of the start codon in 30% of cases. Somatic mutations, on the other hand, occur within the helicase 2 domain [39]. Germline mutations in *DDX41* also differ between ethnic groups. In a study on Korean patients, 10/28 patients were found to harbor p.V152G [40]. In another study, ip.E256K, p.A500fs, p.Y259C, p.E7∗, and p.S363del were the most frequent germline mutations in Japanese patients [38] while approximately 90% of patients harboring p.M1I or p.D140fs mutations were Caucasian [34].

MN with germline mutated *DDX41* is unique as it usually occurs at an older age compared to other MNs with germline mutations. Patients harboring *DDX41* germline mutations develop MDS almost at the same age as the sporadic cases of MDS, with a median age of 65 [35]. Li et al. revealed that AML patients harboring germline mutations have distinct clinicopathologic features including male predominance and an indolent course, and often lack a family history [41]. These findings were later confirmed by Duployez et al. in 2022 [42]. A recent study reported a cohort of patients harboring both germline and somatic *DDX41*, 93% of whom had a normal karyotype. This finding is concordant with other studies, which showed a normal karyotype in approximately 70–80% of cases [30,43]. MNs with germline mutations in *DDX41* have a better OS in comparison to age-matched MDS or AML with wild-type *DDX41* [34,42]. Conversely, Choi et al. demonstrated no significant correlation between *DDX41* mutation and OS [40]. Thus, large multicenter studies are warranted to investigate the relationship between *DDX41* mutation association and overall survival.

There are no randomized studies for MNs with *DDX41* germline mutations; therefore, patients are treated with the same protocols as general MDS/AML patients. Duployez et al. showed that patients with MN with germline *DDX41* have higher complete remission rate when compared to wild-type *DDX41* patients; however, they do not have longer OS [42]. Lenalidomide is a widely recognized standard treatment for low-risk MDS characterized by a deletion of 5q [44]. Lenalidomide has shown good efficacy for MDS/AML patients with *DDX41* mutations in comparison to wild-type *DDX41* patients (100% vs. 53%) [30]. In a prediction model study, lenalidomide showed higher response in patients with MN harboring mutations in the DEAD-box RNA helicase gene, including *DDX41* [45]. Another case report showed successful response to lenalidomide in a patient with high-risk MDS and harboring both germline and somatic mutations in *DDX41* [46]. Currently, there is no consensus for familial screening for patients found to be carriers of *DDX41* germline mutation. Patients harboring germline *DDX41* mutations present with cytopenias at the time of diagnosis [36]. Surveillance with complete blood count (CBC) may be useful in screening carriers of *DDX41* mutations and in early detection of hematologic neoplasms. For the three major pathogenic germline variants in *DDX41* variants (p.D140fs, p.M1I, and p.A500fs) the risk for developing a MN before 40 years of age is negligible; however, increases after 40 [38]. Given the results it might be helpful to start screening at 40 years of age.

*DDX41* mutations also occur in other non-myeloid neoplasms. Recent studies suggest that non-myeloid neoplasms with *DDX41* mutations might have a different pathway from the MN with *DDX41* germline mutations and will not be discussed in this review.

#### 2.1.3. Germline *TP53* P/LP Variant

Tumor protein 53 (*TP53*) is located on chromosome 17p13.1 and encodes p53 protein, which is responsible for cell cycle arrest and acts as an important tumor suppressor. This gene also plays a role in DNA repair and fertility [47]. Li–Fraumeni syndrome (LFS) is a rare autosomal dominant disease caused by germline *TP53* mutations. This syndrome was first described in 1969 by Li and Fraumeni [48]. Germline mutations were first discovered in 1990 [49]. Patients with LFS develop a wide array of hematopoietic malignancies including ALL, AML, and MDS. Patients with this syndrome can also develop early breast cancer, brain cancer, osteosarcoma, and adrenal cortical tumors [50]. The incidence of leukemias (including AML and ALL) in thesepatients is approximately 4% [51]. These leukemias usually develop as a therapy-related neoplasms after radiation therapy and are associated with poor prognosis. There are two criteria established for genetic testing for high-risk LFS cases: classical [52] and Chompret criteria [53]. Carriers of *TP53* germline mutations were found to have a penetrance of 80% by age 70 [54]. A study by Montellier et al. divided *TP53* germline mutations into four classes [55], each with its own unique features.

Class A is identified as patients who carry mutations in the DNA binding domain, especially in the major structural motif of this domain. This class entails all the phenotypic features of a severe LFS picture. Class A is almost identical to carriers of null/frameshift mutations; however, class A tends to have a higher proportion of CNS cancers.

Class B has mutations in the DNA binding domain as well; however, these mutations are present in different locations than class A. These mutations tend to be in positions that are not in direct contact with DNA. Class B mutations exhibit milder functional characteristics and display a broader range of transcriptional activities. These patients have a lower risk of developing cancers, especially in childhood and adolescence. Only partial loss of p53 is sufficient to increase the risk of this cancer.

Class C contains lower penetrance variants that can still cause cancers typical of LFS. This class contains two founder variants: R337H (Brazil) [56] and Y107H hypomorphic variant (African) [57]. The clinical picture of this class is extremely heterogeneous.

Class D represents the low penetrance variants that have strongly attenuated picture of LFS. Carriers of class D variants were more likely to have pathogenic mutations in other cancer predisposition genes than those in the other variant categories.

It is notable that missense *TP53* mutations are the most disease-causing variants as they are associated with a dominant-negative effect (DNE) causing loss of function of p53 tetramers and reducing transcriptional activity of wild-type p53 protein. Immunohistochemistry (IHC) is able to detect cells harboring *TP53* missense mutation and could serve as a surrogate for NGS study.

Myeloid neoplasms with *TP53* germline mutation could be masked by overt phenotypic solid tumor and therapy-induced cytopenia. Peripheral blood monitoring for increased circulating blasts, acquired *TP53* and other gene mutations, and IHC-positive p53 cells in bone marrow core would be helpul for diagnosis. Similar to other *TP53*-mutated tumors, myeloid neoplasm with *TP53* germline mutation also shows poor response to standard therapies and frequently relapses status post allo-HSCT.

Lymphoid neoplasms with *TP53* germline mutation mainly refer to low-hypodiploid B-ALL in children. Clinically it behaves more aggressively and has an adverse clinical outcome. Other types of T- or B-cell lymphomas can be seen with Li–Fraumeni syndrome. Treatment and outcome are similar to myeloid neoplasm with *TP53* germline mutation.

### 2.2. Myeloid Neoplasms with Germline Predisposition and Pre-Existing Platelet Disorders

There is another group of myeloid neoplasms with germline predisposition that presents with platelet disorders. It includes germline *RUNX1* P/LP variant (familial platelet disorder with associated myeloid malignancy), germline *ANKRD26* P/LP variant (thrombocytopenia 2), and germline *ETV6* P/LP variant (thrombocytopenia 5) [58,59,60,61].

#### 2.2.1. Germline *RUNX1* P/LP Variant (Familial Platelet Disorder with Associated Myeloid Malignancy)

Runt-related transcription factor 1 (*RUNX1*) is present on the long arm of chromosome 21 (21q22.12) and plays an important role in ribosome biogenesis [62] and hematopoietic differentiation. There are three different isoforms of *RUNX1* depending on the splice site [63]. Germline mutations in *RUNX1* were first described to be linked to leukemia by Song et al. in 1999 [5]. Later, *RUNX1* familial platelet disorders with associated myeloid malignancies (*RUNX1*-FDPMM) were described by Michaud et al. in 2002 [64], who hypothesized that a secondary mutation is necessary to develop leukemia. This theory was also confirmed by Forster et al. in 2022 [65].

*RUNX1*-FPDMM is inherited in an autosomal dominant manner with incomplete penetrance. The most common presentation in these patients is mild to moderate thrombocytopenia and/or platelet aggregation defects followed by hematologic malignancy [66,67]. The spectrum of mutations in *RUNX1* is vast, including missense, splice site mutations, and large deletions. A recent study by Yu et al. indicated that the most common mutations in these patients resulted in a defective truncated protein via splice site and frameshift mutations. Missense mutations also disrupt *RUNX1* function, affecting important functional domains [68]. The majority of the associated hematologic malignancies affect the myeloid lineage, resulting in MDS and AML; however, cases of T- and B-ALL have been reported [69,70].

Secondary acquired mutations in these patients are diverse. Studies show the most common mutations are *RUNX1* (second allele) and *GATA2*, with an associated decrease in other CHIP mutations [70]. On the other hand, another study found that *BCOR* mutations were the most common ones while other CHIP mutations were frequently present in these patients [68]. Surveillance is difficult since there is no specific phenotype for these diseases. The Kanagal-Shamanna et al. suggested conducting bone marrow biopsies on these patients to establish a baseline picture of the marrow, followed by regular bone marrow biopsies if CBC is abnormal or if NGS shows additional acquired mutations. Differenting between *RUNX1*-mutated AML/ALL and *RUNX1*-FPDMM can be challenging if no clear clinical family history is provided. For sporadic *RUNX1*-mutated AML, the VAF is usually <40%. Notably, the VAF of a mutation can help suggest a mutation is somatic or germline; however, is not definitive. In order to make that distinction, germline testing should be performed on skin fibroblasts or buccal mucosa. The non-germline form is also not associated with inherited platelet defects. Genetic and molecular consultation for family members may provide a clue.

#### 2.2.2. Germline *ANKRD26* P/LP Variant (Thrombocytopenia 2)

Ankyrin repeat domain-containing protein 26 (*ANKRD26*) is located on the short arm of chromosome 10 (10p12.1). It is expressed in many parts of the body, such as the brain and liver, adipose and hematopoietic tissue [71]. This gene plays an important role in cellular differentiation through the formation of more elaborate cell processes [72]. Upregulation of this gene in megakaryocytes causes increased signaling via the thrombopoietin/myeloproliferative leukemia virus oncogene (MPL) pathway and causes dysfunction in proplatelet formation [73].

Inherited thrombocytopenia, linked to chromosome 10p11.1-12, was first described by Savoia et al. in 1999 [74]. Thereafter, two genes (*MASTL* and *ABCD5*) were inaccurately implicated as the cause of this condition [75,76]. In 2011 Pippucci et al. demonstrated that the gene responsible for this condition was *ANKRD26* [77]. *ANKRD26*-related thrombocytopenia is a non-syndromic autosomal dominant condition with increased predisposition to hematologic neoplasms. The most mutated region of *ANKRD26* gene in this disease population is the 19-nucleotide region of the 5′ untranslated region (5′ UTR) (c.-116 through c.-134), which disrupts *RUNX1* and *FLI1* from binding to *ANKRD26*, leading to increased expression of this gene in megakaryocytes [78]. Two other variants involving the 5′ UTR have been reported in *ANKRD26*-related thrombocytopenia: c.-140C > G and c.-113A > C; however, interestingly, both of these variants are also present in the general population [79,80]. Mutations in the 5′UTR or N-terminal domain of *ANKRD26* disrupt its normal suppression, enhancing type I cytokine signaling through MAPK pathway hyperactivation and ultimately leading to altered megakaryopoiesis [81].

There are other variants outside the 5′ UTR region that have been implicated in this disease including a missense mutation involving exon 1 (c.473A > G) and a fusion between the *ANKRD26* and *WAC* genes [82,83]. Dell’Orso et al. described a fusion between *ANKRD26* and *ACBD5,* forming a chimeric protein that also leads to the development of *ANKRD26*-related thrombocytopenia [84].

A large cohort in Italy found that *ANKRD26*-related thrombocytopenia represented 10% of total cases of inherited thrombocytopenia; however, the worldwide incidence cannot be accurately estimated [85]. As for the incidence of MNs in patients with *ANKRD26* germline mutations, it was estimated to be around 8% [86]. The most common MNs in this population are AML and MDS. In comparison to the general population, there is a 24-fold increase in the developing a MN [87].

**Figure 1:**
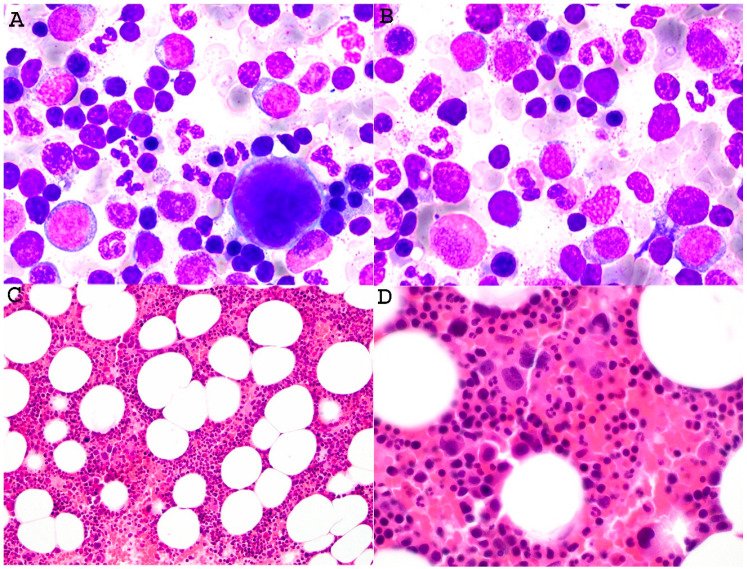
Representative images of bone marrow findings in a 34-year-old patient with *ANKRD26* germline mutation. The bone marrow aspirate displayed dysplastic megakaryocytes with frequently small or hypolobated forms ((**A**), Wright Giemsa, ×1000). Occasional myeloblasts are identified, along with increased hematogones (on average 5.8% in the case) ((**B**), Wright Giemsa, ×1000). The bone marrow core demonstrated slight hypercellularity with identifiable dysplastic appearing megakaryocytes, but no morphologic evidence of abnormal localization of immature precursors. The estimated M:E ratio was 2:1 ((**C**,**D**), H&E, ×200 and ×600, respectively). NGS also detected *ANKRD26* chr10:g.27,389,383 C>T (c.-128 C>T) mutation in the 5′ UTR region of exon 1 in both bone marrow and skin specimens, supporting a diagnosis of *ANKRD26* germline mutation. Additional study demonstrated the patient has familial autosomal dominant *ANKRD26*-mutated thrombocytopenia (exon 1 c-128 G>A). This image is provided courtesy of Dr. Ling Zhang.

Wahlster et al. demonstrated 8 patients with a characteristic phenotype in *ANKRD26*-related thrombocytopenia. Bone marrow examination showed a base-line increase in myeloblasts and dysmegakaryopoiesis (Figure 1) without somatic mutations, and the patients did not develope myeloid neoplasms in long-term follow-ups, indicating a non-malignant process [81]. The potential problem is determining if the dysmegakaryopoiesis is the patient’s baseline or part of a developing MDS. NGS can help establish which are acquired somatic mutations, which would favor a MDS. 

#### 2.2.3. Germline *ETV6* P/LP Variant (Thrombocytopenia 5)

ETS variant transcription factor 6, also known as ETS tranlocation variant 6 (*ETV6*), is located on chromosome 12p and is present in hematopoietic stem cells and progenitor cells. It is an important transcriptional repressor in hematopoietic stem cells [88]. *ETV6* germline mutations are associated with thrombocytopenia and other hematologic malignancies and were first described in 2015 [89,90]. *ETV6* inactivation causes thrombocytopenia due to is effect on megakaryocytes; however, the same inactivation does not affect the B-cell lineage [88]. The peneterance of thrombocytopenia in patients harboring the *ETV6* germline mutation approaches 90%, whereas in hematologic malignancy, it is estimated to be ~20–30% [58]. *ETV6* germline mutations predispose individuals to AML, MDS, and B-ALL, which are the most common malignancies identified [91,92]. Most of the damaging variants were found to be in the ETS DNA-binding domain or cause truncation of this domain. The result of these variants is lost capacity to interact with *ETV6*, target DNA, and achieve proper nuclear localization [92].

The development of leukemia in these patients is dependent on secondary somatic mutations. For example, a patient harboring the *ETV6* R386fs variant developed B-ALL followed by therapy-related AML. On sequencing both leukemias, a *PAX5* mutation was present at the time of the B-ALL and a *CBL* mutation when the patient developed the AML [92]. The median age of hematologic malignancy in patients with *ETV6* germline mutations is 11 years of age, which is older than patients with sporadic *ETV6*-mutated leukemias [93].

### 2.3. Myeloid Neoplasms with Germline Predisposition and Potential Organ Dysfunction

#### Germline *GATA2* P/LP Variant (*GATA2* Deficiency) [94,95]

**Figure 2:**
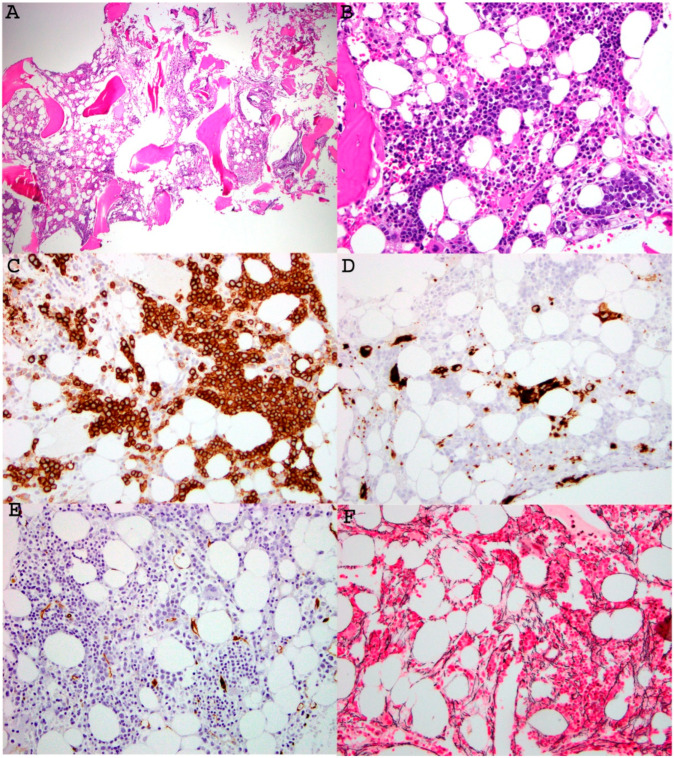
Representative images of bone marrow findings in a 21-year-old patient with MDS associated with germline *GATA2* mutation. A low-power view of the bone marrow core biopsy ((**A**), H&E, ×100) showed patchy fibrosis, decreased myelopoiesis, and atypical/dysplastic megakaryocytes. A medium power of view of the core ((**B**), H&E, ×200) highlighted erythroid preponderance and left-shifted maturation, and hypolobated megakaryocytes. Myeloid precursors were decreased without morphologic evidence of excess myeloblasts. Immunohistochemical stains were performed on the core biopsy, which showed increased CD71 positive erythroid lineage, CD61-positive dysplastic small megakaryocytes, and no increase in CD34 positive blasts ((**C**–**E**), immunoperoxidase, ×200). Reticulin stain highlighted mild reticulin fibrosis (MF1-2/3) ((**F**), reticulin stain, ×200). NGS study confirmed presence of *GATA2*: p.L315Cfs*11, c.942-943delinsC (VAF 49.7%) in both bone marrow and skin specimens, supporting a diagnosis of *GATA2* germline mutation. This image is provided courtesy of Dr. Ling Zhang.

The *GATA2* gene, located on 3q21, is a key transcription factor and plays an essential role in regulation of normal hematopoiesis and lymphangiogenesis. *GATA2* mutations impair hematopoiesis, resulting in defective trilineage differentiation and enhancing over-differentiation in monocytes, eventually leading to myeloid malignancies (Figure 2).

Myeloid neoplasms with germline *GATA2* mutation or *GATA2* deficiency syndrome are a group of diseases secondary to germline mutation involving the *GATA2* gene. The mutation leads to the loss of normal function of the transcriptional factor, *GATA2*, leading to increased risk of MDS and AML with the most common hereditary predisposition to pediatric MDS. These patients often have associated immunodeficiencies, susceptibility to infection, lymphoedema, and *GATA2* deficiency syndromes, e.g., Emberger syndrome, MonoMAC syndrome, DCML deficiency, and WILD syndrome.

Emberger syndrome is very rare autosomomal dominant disorder caused by inactivating mutations of parental *GATA2* genes, which can be heterozygous or homozygous. Patients and family members often manifest with primary lymphedema, hearing loss, and development of MDS and AML.

MonnMAC syndrome is a similar inactivating mutation of the *GATA2* gene can induce a rare autosomal dominant syndrome, characterized by **M**onocytopenia and mycobacterial (**MAC**) infections. Laboratory studies can reveal B-cell lymphopenia, decreased NK cells and dendritic cells. Patients with MonoMAC syndrome often suffer from bacterial, mycobacterial, viral, and fungal infections and develop myeloid neoplasms. Given the profound cytopenia and immunoinsufficiency, the syndrome requires close clinical monitoring to prevent a fatal outcome.

DCML deficiency is characterized by deficiencies of **D**endritic **C**ell, **M**onocyte, B-, and NK-**L**ymphoid cells due to *GATA2* mutation. The mutation results in neutropenia with various infections, in particular mycobacterial infections. Besides MDS and AML, pulmonary alveolar proteinosis and HPV-associated cancers can occur.

WILD syndrome is a rare genetic disorder characterized by **W**arts, **I**mmunodeficiency, **L**ymphedema, and anogenital **D**ysplasia found in patients with a mutated *GATA2* gene.

Diagnosis can be challenging given the number of variants and mutation patterns (germline versus somatic). It is worth mentioning that *GATA2* germline mutations could be a major diagnostic pitfall if only exonic regions are sequenced, missing deletions or variants in intronic regions [96]. The majority of routine NGS myeloid panels do not sequence intron regions. Thus, whole genome sequencing is necessary to detect such variants if there is high clinical suspicion. The curative treatment strategy is allogeneic hematopoietic stem cell transplant.

### 2.4. MNGPs with Potential Organ Dysfuntion Also Include Dyskeratosis Congenita and Down Syndrome

#### 2.4.1. Dyskeratosis Congenita [97,98]

Dyskeratosis congenita (DC) is the prototypical telomere biology disorder clinically presenting with a triad of oral leukoplakia, nail dystrophy, and reticular hyperpigmentation. It occurs in childhood or adolescence and is commonly inherited in an X-linked pattern; however, AD or AR patterns have also been reported. There are at least 18 germline mutations (coding, splicing, and deletions) (e.g., *ACD*, *CTC1*, *DKC1*, *MDM4*, *RTEL1*, *TERC*, *TERT*, *TINF2*, *NHP2*, *NOP10*, *NPM1*, *PARN*, and *WRAP53*). *DKC1* is the most common mutation. These genes are critical for telomere maintenance. Patients with DC are at a high risk for inflammatory fibrosis, pulmonary fibrosis, liver cirrhosis, vascular anomalies, squamous cell carcinoma, aplastic anemia, or MDS associated with monosomy 7. Bone marrow failure is one of the common findings. Diagnosis of DC is dependent on clinical findings and detection of germline mutations associated with telomere biology. Telomere length measurement also helps detect telomere biology disorders. Positive family history requires genetic consultation and comprehensive investigation. Treatment is challenging. Allo-HSCT is probably the best approach for DC patients who develop bone marrow failure with or without MNs.

#### 2.4.2. Down Syndrome [99,100,101,102,103,104,105,106]

**Figure 3:**
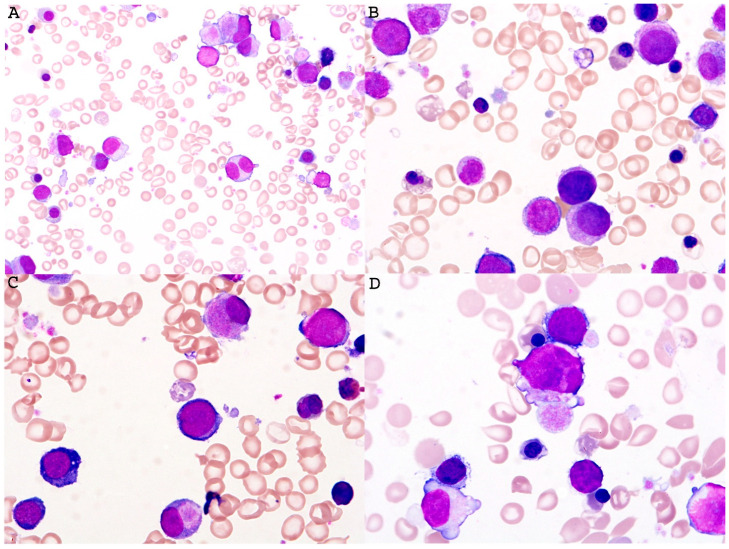
Representative images of Down syndrome-associated transient abnormal myelopoiesis (TAM) in an infant’s peripheral blood smear. The smear (**A**–**D**) showed marked leukoerythroblastosis including numerous nucleated red blood cells and circulating micromegakaryocytes, myeloblasts, and megakaryoblasts. Background platelets showed anisopoikilocytosis including many giant forms (Wright stain, (**A**), ×200, and (**B**–**D**), ×1000, respectively). This image is provided courtesy of Dr. Ling Zhang.

Down syndrome represents the most prevalent chromosomal aneuploidy observed in neonates. Myeloid neoplasms in Down syndrome (DS) represent a unique spectrum of diseases that differ significantly from leukemia in the general population. They typically follow two well-defined patterns: (1) transient abnormal myelopoiesis (TAM) and (2) myeloid leukemia associated with Down syndrome (ML-DS). The former presents within the first three weeks of birth and shows leukoerythroblastosis with giant platelets and megakaryoblasts in the blood (Figure 3) and undergo spontaneous remission within 3 months with no need for treatment; however, around a quarter of these patients will develop acute leukemia or MDS. The second group develops MDS or AML with megakaryoblastic differentiation, which requires appropriate treatment according to the therapeutic guideline for ML-DS.

Patients with Down syndrome also have an increased risk of developing ALL. ALL in Down syndrome (DS-ALL) has a unique biology, frequently presenting with *CRLF2* mutation, and occurs more commonly in this patient population compared to the general population. The most frequent genetic abnormality is B-ALL with *CRLF2* rearrangement, which accounts for 50–60% of cases as compared to 5% in the non-DS population. Almost half of patients with *CRLF2* rearrangement also harbor an additional *JAK2* mutation.

The pathogenetic mechanism is a combination of trisomy 21 and acquired *GATA1* mutation, which results in dysregulated hematopoiesis. The development of these myeloid neoplasms is a classic example of multi-step leukemogenesis, as the presence of an extra chromosome 21 disrupts fetal hematopoiesis by expanding the pools of hematopoietic stem cells and megakaryocyte–erythroid progenitors, while simultaneously suppressing the development of B-cell precursors. The presence of the *GATA1* mutation is a mandatory first step toward ML-DS, but it is not sufficient to cause disease on its own. VAF demonstrates a highly significant positive correlation with blast percentage, leukocyte counts, and morphological markers of dysplasia (specifically dyserythropoiesis and dysmegakaryopoiesis). The size of the *GATA1* clone at birth was the sole factor capable of predicting future ML-DS. While most *GATA1* clones identified in newborns do not develop into leukemia, any clone that remains detectable for more than six months is at a significant risk of leukemic transformation. To develop myeloid leukemia in Down syndrome, additional acquired somatic mutations are needed. In addition to *GATA1* mutations, ML-DS is commonly driven by acquired mutations in *CTCF*, cohesin genes, and the RAS or JAK/STAT pathways.

An extra chromosome 21 can be detected in all cells (trisomy 21) or in some cells (mosaic) during conventional karyotyping. Presence of chromosomal abnormality leads to developmental issues and phenotypic changes, including intellectual disability, facial features (widened eyes, flattened nasal bridge, low-set ears), joint laxity, and muscular hypotonia. Trisomy 21 patients often present with variable degrees of organ defects involving cardiac, endocrine, and neurosensory systems and gastrointestinal tract. Diagnosis relies on karyotyping and NGS studies. There are about 10% of infants with TAM with severe complications leading to multiorgan failure. The risk of TAM transforming to acute megakaryoblastic leukemia is approximately 20–30% between 3 months and 3 years of age. Close clinical monitoring is recommended as low-dose chemotherapy can reduce mortality in patients who develop acute leukemia.

### 2.5. Germline SAMD9 Mutations

Variant mutations include (1) germline *SAMD9* P/LP variant (MIRAGE syndrome), (2) germline *SAMD9L* P/LP variant (*SAMD9L*-related ataxia–pancytopenia syndrome), and (3) biallelic germline *BLM* P/LP variant (Bloom syndrome).

#### 2.5.1. Germline *SAMD9* P/LP Variant (MIRAGE Syndrome)

The sterile alpha motif domain containing 9 (*SAMD9*) is located on chromosome 7q21.2 and is involved in antiviral factors and tumor suppression. It plays an important role in the development of different MNs [107] and is responsible for the development of normophosphatemic familial tumoral calcinosis. This condition has been reported in Jewish Yemenite families with biallelic loss-of-function mutations in *SAMD9* (p.K1495E and p.R344X) [108,109]. Germline mutations in *SAMD9* can lead to the development of MIRAGE syndrome (myelodysplasia, infection, growth restriction, adrenal hypoplasia, genital anomalies, and enteropathy), which was first discovered in 2016 [110]. Interestingly, haploinsufficiency of *SAMD9* has also been implicated in the development of MDS [111]. MIRAGE syndrome develops due to a gain-of-function mutation in the *SAMD9* gene. In cases of gain-of-function mutations in *SAMD9*, the body can develop a defense mechanism by either by losing the mutated allele, also known as adaptation by aneuploidy, or it can develop a second hit somatic mutation within the *SAMD9* gene, leading to the reversal of the mutation [112].

*SAMD9* germline mutations have been implicated in the development of a large proportion of pediatric MDS cases, ranging from 8–17% [113,114]. Similarly, a study in France showed that approximately 18% of children with idiopathic cytopenias harbored *SAMD9/9L* mutations [115]. *SAMD9* germline mutations are usually missense mutations. The germline mutations associated with MIRAGE syndrome tend to cluster around the P-loop NTPase domain; however, MDS cases with *SAMD9* mutations occur across the protein domain structure [116]. On the other hand, another study by Narumi et al. suggests that there is no association or particular clustering [117]. De novo acquired mutations causing MIRAGE syndrome exhibit complete penetrance [118]. Many patients with MIRAGE syndrome do not exhibit the whole phenotypic spectrum of the disease. Around 20% of children with MIRAGE syndrome do not exhibit adrenal symptoms, tend to be heavier in weight at birth, and have a longer gestational period compared to their counterparts with adrenal disease. These children suffer from various endocrinopathies such as panhypopituitarism and hypothyroidism. Thrombocytopenia is the most common cytopenia present in children with *SAMD9* mutations. The median age of development of pediatric MDS is 9.6 years of age [118]. The most common cytogenetic abnormality in these patients is monosomy 7 [115,119]. Some studies show that monosomy 7 can be transient [120] and another study showed that certain patients harboring *SAMD9/SAMD9L* and monosomy 7 can undergo spontaneous remission. Thus, close surveillance of these patients may be the preferred approach over upfront stem cell transplant [121]. Adult onset MDS with *SAMD9* mutation occurs in around 3% of MDS patients and is usually a loss-of-function rather than gain-of-function mutation, which is typically seen in pediatric patients [122]. Another condition caused by germline mutations in *SAMD9*, *SAMD9L* as well, is monosomy 7 myelodysplasia and leukemia syndrome 2 (M7MLS2). This condition follows an autosomal dominant pattern of inheritance [123] and has variable manifestations ranging from cytopenias to frank MDS and AML [123,124].

#### 2.5.2. Germline *SAMD9L* P/LP Variant (*SAMD9L*-Related Ataxia–Pancytopenia Syndrome)

Sterile alpha motif domain-containing protein 9-like (*SAMD9L*) is a gene present on chromosome 7. It is a tumor suppressor gene and plays a role in innate immunity against viral infections such as poxviruses [107,125]. Germline gain-of-function mutations in this gene cause ataxia–pancytopenia syndrome. This syndrome was first described by Frederick Li, who also helped discover Li–Fraumeni syndrome [126]. A newly discovered germline variant (S1473N) was found to play a role in the development of this disease [127]. This syndrome spans neurological (cerebellar ataxia) and hematological symptoms (varying cytopenias). S1473N predisposes one to hematologic conditions, including leukemia and complete marrow failure, and similar to MIRAGE syndrome, monosomy 7 has been described in this condition. Ataxia–pancytopenia syndrome follows an autosomal dominant pattern of inheritance [128]. Given the variable clinical presentation of this syndrome, the estimated penetrance is not known; however, it has been found that most patients carrying mutations in *SAMD9L* express some features of this syndrome. It has been reported that patients harboring *SAMD9L* mutations can undergo revertant mosaicism to abrogate the effect of gain-of-function mutations in *SAMD9L*. This happens either by loss of the 7q arm or through cis-truncating mutations that abolish the gain-of-function of the mutant allele [129]. Whether this somatic rescue mechanism increases the risk of development of myeloid neoplasms later in life is dependent on the mechanism. For example, uniparental disomy rescue increases the risk of developing of myeloid neoplasms [119]. On the other hand, somatic rescue occurring through a cis-truncating mutation in *SAMD9L* increases the potential for developing a myeloid neoplasm later in life because it results in haploinsufficiency of *SAMD9L* [122]. Germline mutations of *SAMD9L* can also cause myeloid neoplasms in adults; however, these mutations are usually missense or nonsense mutations causing loss-of-function mutations in contrast to the gain-of-function mutations that occur in the pediatric population [130]. In a recent study in 2023, 2% of adult patients with hypocellular bone marrow harbored germline mutations in *SAMD9*/*SAMD9L* [131]. *SAMD9L* germline mutation displays incomplete penetrance with varying manifestations ranging from pancytopenia to MDS or AML [124]. *SAMD9L*-associated autoinflammatory disease, caused by truncating mutations in *SAMD9L*, is an acute, early-onset inflammatory syndrome characterized by skin nodules (panniculitis) and lung disease (interstitial inflammation) driven by neutrophils. It causes profound systemic inflammation, as indicated by significantly elevated C-reactive protein alongside an immunodeficiency marked by the progressive loss of B- and NK cells [132].

#### 2.5.3. Biallelic Germline *BLM* P/LP Variant (Bloom Syndrome)

Bloom syndrome is caused by mutations in the Bloom syndrome helicase *(BLM*) gene, located on chromosome 15. This syndrome has high prevalence in the Ashkenazi Jewish population [133]. The *BLM* helicase is a protein that belongs to the RecQ family and is essential for processes like DNA repair and proper cell cycle progression. Its failure leads to genomic instability, which explains the syndrome’s defining feature: a significant predisposition to various malignancies [134]. Patients with Bloom syndrome have a spectrum of growth deficiency and clinically manifest with increased sun sensitivity and susceptibility to infections due to immunodeficiency. The most common neoplasms that occur in the first two decades of life in these patients are hematolymphoid neoplasms [135]. Additionally, colorectal cancer also frequently occurs in these patient at a younger age. The syndrome follows an autosomal recessive inheritance pattern.

This syndrome usually occurs as a result of loss-of-function mutations, which leads to chromosomal instability, drives excessive homologous recombination, and dramatically raises the frequency of sister chromatid exchanges [136]. Diagnosis can be made according to positive family history, clinical presentation, and detection of *BLM* gene mutations. Treatment is supportive including prevention of infection, sun protection, early cancer surveillance, and monitoring for insulin resistance. So far, the disease is not curable given the genetic defect.

## 3. Conclusions

Following deep sequencing implications in the daily diagnosis of benign and malignant hematologic disorders, emerging germline mutations likely driving myeloid neoplasms have been identified. They include the germline *CSF3R*, *ERCC6L2*, *JAK2*, *MBD4*, *MECOM*, *NPM1*, *RBBP6*, *SRP72*, and *TET2* P/LP variants. Attention should be paid to these gene mutations when identified in younger patients or those who have a familial history of hematological malignancy.

It is important to follow patients who carry germline mutations to monitor for hematologic disease. A recent study in 2023 proposed certain features that are associated with progression of patients with germline predisposition to myelodysplastic syndrome.

Increased blasts in bone marrow (≥5%) or peripheral blood (≥2%) or two of the following features:

• Acquired somatic pathogenic mutation;
• New cytopenia in a different lineage or progressive cytopenia in the same lineage, especially in the context of increased bone marrow cellularity;
• Multilineage dysplasia;
• MDS defining cytogenetic or molecular abnormalities.

These features help determine which population of patients should be monitored more closely. The dysplasia in these patients should be monitored carefully, and a bone marrow biopsy should be done to establish the patient’s baseline morphology and monitor progression of dysplasia if present. Individuals with germline mutations in genes like *RUNX1*, *ANKRD26*, and *ETV6* often start with low platelet counts and show abnormal megakaryocytes in their bone marrow. Because these features are expected consequences of the inherited mutation, pathologists must be careful, as the presence of megakaryocytic atypia alone is insufficient to diagnose MDS in these patients [137].

There is no consensus on how to monitor or follow up patients with myeloid neoplasms and germline predisposition. Periodic bone marrow examination is important for monitoring any deviation from the patient’s baseline bone marrow, which can change in cellularity or dysplasia. It is imperative to avoid overdiagnosing these patients with progression to MDS [138]. The only curative option for patients who develop myeloid neoplasms is stem cell transplant; however, patients who develop high-grade myeloid neoplasms, such as AML, are not responsive to conditioning chemotherapy, which complicates the process.

## 4. Future Directions

There is still a lot unknown regarding hematologic disorders associated with germline mutations. The 5th edition of WHO and ICC delineated a few new emerging germline genetic disorders that are likely associated with myeloid neoplasms. The germline *CSF3R* mutation, for example, can cause severe congenital neutropenia, multiple myeloma, or B-ALL [139,140]. There are many other conditions that still need to be investigated. Implementing NGS is of imperative importance to discover these entities and help reach an earlier diagnosis.

## Figures and Tables

**Table 1:** Myeloid neoplasms with germline predisposition and other inherited syndromes (WHO and ICC).

4th ed. WHO	5th ed. WHO	ICC	Clinical Presentation	Associated Diseases
**Myeloid neoplasms with germline predisposition (MNGPs)**	**Myeloid neoplasms associated with germline predisposition (MNGPs)**	**Hematologic neoplasms associated with germline predisposition (HNGPs)**		
**MNGPs without a pre-existing disorder or organ dysfunction**	**MNGPs without a pre-existing platelet disorder or organ dysfunction**	**HNGPs without a constitutional disorder**		
AML with germline *CEBPA* mutation	Germline *CEBPA* P/LP variant (*CEBPA*-associated familial AML)	MN with germline *CEBPA* mutation		*CEBPA*: AML, familial AML with mutated *CEBPA*
MN with germline *DDX41* mutation	Germline *DDX41* P/LP variant	M/LN with germline *DDX41* mutation		*DDX41*: MDS and AML, familial AML with *DDX41* mutation
	Germline *TP53* P/LP variant (Li–Fraumeni Syndrome)	M/LN with germline *TP53* mutation (Li-Fraumeni syndrome)		*TP53*: hypoplastic ALL, t-MN, solid tumors, Li–Fraumeni syndrome
**MNGPs with pre-existing platelet disorder**	**MNGPs with pre-existing platelet disorder**	**HNGPs associated with a constitutional platelet disorder**		
MN with germline *RUNX1* mutation	Germline *RUNX1* P/LP variant (familial platelet disorder with associated myeloid malignancy)	M/LN with germline *RUNX1* mutation	*RUNX1:* Thrombocytopenia, decreased platelet function	*RUNX1*: MDS, AML, infrequently T-ALL, familiar platelet disorders with propensity for myeloid neoplasm
MN with germline *ANKRD26* mutation	Germline *ANKRD26* P/LP variant (thrombocytopenia 2)	MN with germline *ANKRD26* mutation	*ANKRD26:* Thrombocytopenia, decreased platelet function	*ANKRD26*: MNs
MN with germline *ETV6* mutation	Germline *ETV6* P/LP variant (thrombocytopenia 5)	M/LN with germline *ETV6* mutation	*ETV6:* Thrombocytopenia, decreased platelet function	*ETV6*: MDS, AML, ALL, thrombocytopenia 5
**MNGPs associated with other organ dysfunction**	**MNGPs with potential organ dysfunction**	**HNGPs associated with a constitutional disorder affecting multiple organ systems**		
MN with germline *GATA2* mutation	Germline *GATA2* P/LP variant (*GATA2* deficiency)	MN with germline *GATA2* mutation	*GATA2:* Immuno-deficiency, monocytopenia, B-cell lymphopenia	*GATA2*: MDS, AML, Emberger syndrome, and MonoMAC syndrome
	Germline *SAMD9* P/LP variant (MIRAGE syndrome)	MN with germline *SAMD9* mutation	*SAMD9*: Bone marrow failure	*SAMD9*: MDS, AML with monosomy 7, MIRAGE syndrome, myelodysplasia/leukemia syndrome with monosomy 7
	Germline *SAMD9L* P/LP variant (*SAMD9L*-related ataxia–pancytopenia syndrome)	MN with germline *SAMD9L* mutation	*SAMD9L*: Systemic auto-inflammatory disease, bone marrow failure	*SAMD9L*: ataxia-pancytopenia syndrome, myelodysplasia and leukemia syndrome with monosomy 7
	Biallelic germline *BLM* P/LP variant (Bloom syndrome)		Prenatal growth deficiency Mild immunodeficiency Profound photosensitivity Type II diabetes mellitus Hypogonadism	
**MNGPs associated with inherited bone marrow failure syndromes**	**Bone marrow failure syndromes**	**MNs associated with bone marrow failure syndromes**		
Fanconi anemia	Fanconi anemia	Fanconi anemia		
Severe congenital neutropenia	Severe congenital neutropenia	Severe congenital neutropenia		
Shwachman–Diamond syndrome	Shwachman–Diamond syndrome	Shwachman–Diamond syndrome		
Diamond–Blackfan anemia		Diamond–Blackfan anemia		
**MNGPs associated with telomere biology disorders**	**Telomere biology disorders**	**Telomere biology disorders**		
Dyskeratosis congenita	Dyskeratosis congenita	Dyskeratosis congenita		
Syndromes due to *TERC* or *TERT* mutations	Syndromes due to *TERC* or *TERT* mutations			
**NA**	**RASopathies**	**NA**		
Previously described in JMML and MDS sections	Neurofibromatosis type 1 CBL syndrome Noonan syndrome Noonan syndrome-like disorders	Included in pediatric disorders and/or germline mutation-associated disorders	RAS-MAPK pathway: JMML-like proliferation with spontaneous regression	RAS-MAPK pathway: JMML, ALL, AML, Noonan syndrome, RASopathy, Neurofibromatosis type 1
**Myeloid proliferations associated with Down syndrome**				
Transient abnormal myelopoiesis associated with Down syndrome	Transient abnormal myelopoiesis associated with Down syndrome	Transient abnormal myelopoiesis associated with Down syndrome		
Myeloid leukemia associated with Down syndrome	Myeloid leukemia associated with Down syndrome	Myeloid leukemia associated with Down syndrome		

ALL: acute lymphoblastic leukemia; AML: acute myeloid leukemia; HNGPs: Hematologic neoplasms associated with germline predisposition; ICC: International Consensus Classification; JMML: juvenile myelomonocytic leukemia; HNGPs: Hematologic neoplasms associated with germline predisposition; M/LN: myeloid/lymphoid neoplasm; P/LP variant: pathologic/likely pathologic variant; WHO: World Health Organization.

## Data Availability

The original contributions presented in this study are included in the article. Further inquiries can be directed to the corresponding author.
